# Effect of organic and inorganic manganese supplementation on performance and eggshell quality in aged laying hens

**DOI:** 10.1002/vms3.1116

**Published:** 2023-03-15

**Authors:** Heydar Zarghi, Ahmad Hassanabadi, Nafise Barzegar

**Affiliations:** ^1^ Department of Animal Science Faculty of Agriculture Ferdowsi University of Mashhad Mashhad Iran

**Keywords:** bio‐efficacy, laying hen, manganese requirement, manganese source, regression model

## Abstract

**Background:**

Manganese (Mn) is an important trace element for laying hen's nutrition, which is required in small amounts in the diet. Its deficiency results in lowered production performance and eggshell quality.

**Objectives:**

This experiment was conducted to investigate the influence of different sources and levels of Mn on egg production performance and eggshell quality in aged laying hens.

**Methods:**

A total of 720, 83‐week‐old Hy‐Line W‐36 laying hens were fed a non‐Mn supplemented basal diet for 4‐week (to ime Mn‐exhaustion of body) and then were allocated to a completely randomized design with 10 treatments, six replicates and 12 birds each. Concentration of Mn in the non‐Mn supplemented basal diet was 10.34 mg/kg (treatment 1), the added doses of dietary Mn were included 30, 60, and 90 mg/kg of three different sources (Mn‐oxide, Mn‐sulphate, and Mn‐organic) for treatments 2‐10, respectively. The experiment lasted for 12 week.

**Results:**

Dietary supplementation with either organic or inorganic Mn sources significantly enhanced egg production (EP), egg mass (EM), feed conversion ratio (FCR), and relative eggshell weight (RESW) compared with the non‐Mn supplemented diet. However, the experimental diets did not influence feed intake (FI), egg weight, and other eggshell quality traits. Based on the broken line regression models, the performance traits were optimized at 30–40 mg/kg Mn concentration when supplemented by Mn‐sulphate or Mn‐organic. Although, it was 80–90 mg/kg when supplemented by Mn‐oxide. The relative bio‐efficacy of inorganic Mn sources include Mn‐oxide and Mn‐sulphate in compare with Mn‐organic were estimated 45% and 87% (for EP trait), 30% and 94% (for EM trait), 36% and 99% (for FCR trait), and 37% and 78% (for RESW trait), respectively.

**Conclusions:**

In the aged laying hens, Mn requirement is higher than the NRC's recommendation. Sulphate and organic sources of Mn are more effective than Mn‐oxide.

## Graphical abstract

In the aged laying hens: Mn requirement is higher than the NRC (1994) recommendation. Mn‐organic and Mn‐sulphate sources of manganese are more effective than Mn‐oxide.

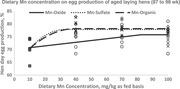

## 1 INTRODUCTION

Since the thin eggshell and broken and cracked eggs are the main problem in the egg production industry, the eggshell quality improvement can be one of the main issues in laying hen farms (Manangi et al., 2015). The percentage of broken or cracked eggs increases with increasing laying hens age and egg size (Khatibi et al., 2021). Mineral nutrition is a remarkable side for optimum egg production and quality in commercial layers. Deficiency of minerals is one of the influential factors responsible for eggshell disorders in laying hens. The saving of optimum mineral concentration in the diets is crucial for getting high‐quality eggs for sale (Saleh et al., 2020). Trace minerals are substantial in poultry nutrition due to their role in various biochemical processes needed for normal growth (Zarghi et al., 2022), maturation, and eggshell deposition (Saleh et al., 2020).

Manganese (Mn) is an important trace element for laying hen's nutrition, which is required in small amounts in the diet (Olgun, 2017). Mn is a cofactor for a wide range of metalloenzymes. It is utilized by almost all forms of life (Culotta et al., 2005). It is also involved in skeletal system development, energy metabolism, nervous system function, immune system function, and reproductive hormone function (Santamaria, 2008). Mn functions as essential accessory factors to enzymes involved in the synthesis of cholesterol, the precursor for the production of steroid hormones and essential for proper reproductive function (Saleh et al., 2020). It is also a component of the principal mitochondrial antioxidant enzyme (Abreu & Cabelli, 2010; Johnson & Giulivi, 2005). Mn deficiency results in lowered egg production and reduced eggshell strength (Lu et al., 2007; Suttle, 2010). Further, the eggshell ultrastructure becomes abnormal, especially in the morphology of mammillary knobs (Xiao et al., 2015).

However, the National Research Council had recommended a dietary Mn concentration of 20–25 mg/kg for laying hens (NRC, 1994). This suggested concentration may no longer be sufficient to maintain the optimal performance and eggshell quality of laying hens due to the considerable improvement in their egg production (Olgun, 2017). Additionally, the reported Mn requirement may vary with the type of diet and supplement used. In the recent diets, the Mn requirement is considered higher than these values (mHy‐Line, 2020; Olgun, 2017).

Minerals are added to the poultry diets in inorganic or organic forms. In organic form, minerals linked with amino acids and become stable and more bioavailable (Saleh et al., 2018). Commercial diets are typically supplemented with inorganic trace minerals at a proportion greater than the recommendation (Saleh et al., 2020). Meanwhile, use of inorganic trace minerals holds several risks including the formation of insoluble complexes, low digestibility, and environmental pollution via their excretion in urine and faeces (Zarghi et al., 2022). The bioavailability of Mn is very low in most practical feedstuffs, and there is evidence that practical ingredients reduce the bioavailability of inorganic dietary Mn (Halpin & Baker, 1986). Previous results showed that the absorption rate of organic minerals in the gut was greater than it was in inorganic form (Saleh & El‐Magd, 2018). Some studies reported that organic Mn sources have more positive effects on performance (Klecker et al., 2002) and eggshell quality (Klecker et al., 2002; Xiao et al., 2015) than the inorganic sources. In contrast, other studies indicate no difference between organic and inorganic Mn sources (Lim & Paik, 2003; Mabe et al., 2003). Given this background, this study was undertaken to evaluate the effects of different levels of dietary Mn supplementation of organic (Mn‐amino acid) and inorganic (Mn‐oxide and Mn‐sulphate) sources on egg production performance and eggshell quality of aged laying hens. In addition, it was hypothesized that it is possible to find an optimal dietary Mn concentration and evaluate the relative bio‐efficacy of inorganic to organic Mn sources in aged laying hens.

## 2 MATERIALS AND METHODS

### 2.1 Feedstuffs analysis

Before the experiment, the basal ingredients such as corn (data on as fed basis; dry matter, 89.01%; gross energy, 3959 kcal/kg; crude protein, 7.5%; ether extract, 4.5%; crude fiber, 2.6%; ash, 1.3%) and soybean meal (data on as fed basis; dry matter, 88.52%; gross energy, 4155 kcal/kg; crude protein, 44.1%; ether extract, 2.2%; crude fiber, 2.8%; ash, 5.4%) were analyzed for composition by the methods described (AOAC, 2002), and corn (data on as fed basis; metabolizable energy, 3363 kcal/kg; digestible Lysine, 0.21%; digestible Methionine, 0.17%; digestible sulphur amino acids, 0.32%; digestible threonine, 0.26%) and soybean meal (data on as fed basis; metabolizable energy, 2245 kcal/kg; digestible Lysine, 2.51%; digestible Methionine, 0.57%; digestible sulphur amino acids, 1.12%; digestible threonine, 1.52%) were determined by near‐infrared spectroscopy (Amino NIR) through Evonik Co. (Evonik Nutrition & Care GmbH) agent in Tehran, Iran. These values were us ed for experimental diets formulation.

### 2.2 Housing, birds, and diets

A 16‐week experiment was carried out with 720, 83‐week‐old Hy‐Line W‐36 laying hens. Laying hens had been moved into the house, and four hens were housed in a cage ‘40 × 45 cm, 450 cm^2^/hen’. The experimental period included a 4‐week fed a non‐Mn supplemented ration, Mn‐exhaustion phase, followed by a 12‐week fed experimental diet and data collection phase. The birds were individually weighed and randomly assigned to 10 treatments in a completely randomized design with six replicates/treatment and 12 birds/replicate. The basal diet was formulated to meet energy and nutrients requirements, except Mn, as recommended by the strain Management Guide (mHy‐Line, 2020) in the late phase of the first laying cycle based on 110 g/b daily feed intake on a least‐cost equation by user‐friendly feed formulation done again (UFFDA, 1992) software. The basal diet contained 10.3 mg/kg Mn as fed basis of raw materials (measured by atomic absorption spectrometry analysis). Ingredients and diet compositions are presented in Table 1. All experimental diets were provided in a mash form. The experimental diets were provided in a way that a batch of the basal diet (without Mn supplementation) was made and then divided into 10 equal portions; the definite dosage of Mn (inorganic Mn supplements as reagent‐grade Sigma–Aldrich chemical Co., St. Louis, ‘Mn‐oxide: Mn_2_O_3_, 69.62% Mn’ at the rate of 43, 86, and 130 mg/kg, or ‘Mn‐sulphate: MnSO_4_.H_2_O, 36.4% Mn’ at the rate of 82, 164, and 247 mg/kg) or organic supplement ‘Availa Mn; Manganese Amino Acid complex 10% Mn, Zinpro Amino Acid Nutrition Inc., Eden Prairie Mn’ at the rate of 300, 600, and 900 mg/kg was added on top of each portion and mixed to make one non‐Mn supplemented diet that contained 10.3 mg/kg Mn concentration and nine experimental diets with 40, 70, and 100 mg/kg Mn concentration supplemented by each source, respectively (Table 2). Birds were reared in an environmentally controlled house with temperature maintained at approximately 18–22°C, and relative humidity was 40%–50%. The lighting program was 16 h light and 8 h darkness. All birds had free access to feed and water throughout the experimental period.

**TABLE 1:** Ingredients and nutrients composition of the basal diet^a^.

Ingredients, g/kg as fed basis	
Corn (ME = 3373 kcal/kg, CP = 7.5%)	641.0
Soybean meal (ME = 2240 kcal/kg, CP = 44%)	216.3
Soybean oil (ME = 8800 kcal/kg)	16.6
Limestone (Ca = 38%)	95.4
Dicalcium phosphate (Ca = 22%, P = 18.7%)	20.7
Common salt	3.5
Vitamin premix^b^	2.5
Mineral premix^c^	2.5
DL‐Methionine	1.4
l‐Lysine HCL	0.1

Calculated nutrients composition^d^, as fed basis	
Metabolizable energy, kcal/kg	2800
Crude protein, %	14.42
Calcium, %	4.14
Phosphorus (available), %	0.48
Sodium, %	0.17
Digestible lysine, %	0.67
Digestible methionine, %	0.35
Digestible sulphur amino acids, %	0.56
Digestible threonine, %	0.47
Manganese, mg/kg	10.30

Abbreviations: Ca, calcium; CP, crude protein; ME, metabolizable energy; P, phosphorus.

^a^ The experimental diets were provided in a way that a batch of basal diet (without Mn supplementation) was made and then divided into 10 equal portions, the definite dosage of Mn (inorganic Mn supplements as reagent‐grade Sigma–Aldrich Chemical Co., St. Louis, ‘Manganese oxide: Mn_2_O_3_, 69.62% Mn’ at the rate of 43, 86, and 130 mg/kg, or ‘Manganese sulphate: MnSO_4_.H_2_O, 36.4% Mn’ at the rate of 82, 164, and 247 mg/kg, or organic supplement ‘Availa Mn; Manganese Amino Acid complex 10% Mn, Zinpro Amino Acid Nutrition Inc., Eden Prairie Mn’ at the rate of 300, 600, and 900 mg/kg) was added on top of each portion and mixed to make 1 ‘non‐supplemented’ contained 10 mg/kg Mn and nine experimental diets with 40, 70, and 100 mg/kg Mn concentration supplemented by each source, respectively.

^b^ Vitamin premix supplied the following per kilogram of diet. vitamin A (all‐trans‐retinol), 4400 IU; vitamin D3 (cholecalciferol), 1000 IU; vitamin E (α‐tocopherol), 11 IU; vitamin K3 (menadione), 2.33 mg; vitamin B1 (thiamin), 2.97 mg; vitamin B2 (riboflavin), 4.4 mg; vitamin B3 (niacin), 22 mg; vitamin B5 (pantothenic acid), 10 mg; vitamin B6 (pyridoxine), 4.45 mg; vitamin B9 (folic acid), 1.9 mg; vitamin B12 (cyanocobalamin), 0.011 mg; vitamin H2 (biotin), 0.18 mg; choline chloride, 487.5 mg, antioxidant 1.0 mg.

^c^ Mineral premix supplied the following per kilogram of diet. Zn (zinc oxide), 75 mg; Mn (manganese oxide), 0.0 mg; Fe (iron sulphate), 75 mg; Cu (copper sulphate), 5 mg; I (ethylene diamine dihydroiodide), 0.76 mg; Se (Sodium Selenite), 0.1 mg; choline chloride, 474 mg.

^d^ Determined ingredient analysis was used to calculate nutrient composition. Crude protein, calcium and sodium were measured by the AOAC (2002) methods; metabolizable energy, digestible amino acids and available phosphorus were measured by NIR analysis.

**TABLE 2:** Manganese supplementation (different source and level) and determined Mn concentration in the experimental diets, as fed basis.

Mn supplementation, mg/kg	Theoretical Mn supplementation, mg/kg	Diets analyzed Mn concentration^d^, mg/kg
Non‐supplemented	0.0	10.4
Mn‐oxide^a^		
43	30	40.20
86	60	70.75
130	90	100.60
Mn‐sulphate^b^		
82	30	40.25
164	60	70.70
247	90	100.25
Mn‐organic^c^		
300	30	40.60
600	60	70.20
900	90	100.70

^a^ Mn_2_O_3_, 69.62% Mn, as reagent‐grade Sigma–Aldrich chemical Co., St. Louis.

^b^ MnSO_4_.H_2_O, 36.4% Mn, as reagent‐grade Sigma–Aldrich chemical Co., St. Louis.

^c^ Manganese Amino Acid complex 10% Mn, Zinpro Amino Acid Nutrition Inc., Eden Prairie Mn.

^d^ Values based on chemical analysis of triplicate samples of diets.

### 2.3 Egg production performance

Egg production (number and weight) and mortality were recorded daily, egg weight was recorded weekly, and feed intake and egg quality traits were recorded monthly. Feed intake was calculated by weighing the residual feed in feeders for the respective period. Feed consumption was adjusted for mortality. The feed conversion ratio (FCR) was adjusted for mortality and calculated as follows: total feed intake divided by total egg mass.

### 2.4 Egg quality

For evaluating egg quality traits, six eggs/replicate (36 eggs/treatment) from eggs laid throughout the 3 consecutive days by the end of every period (26–28 days) were randomly selected and transported to an egg quality laboratory. On the sampled eggs, maximum width and length were measured by passing the eggs through the digital Caliper (0.05 mm, Model 1116−150, Insize Co. Ltd., Suzhou, China) to obtain the maximum points and then calculating the egg shape index by the following formula (Hossaninejad et al., 2021):

$$\mathrm{Shape}\;\mathrm{index}\;=\frac{\mathrm{Egg}\;\mathrm{width}}{\mathrm{Egg}\;\mathrm{length}}\;\;\times 100.$$

After weighing individual eggs with a digital electronic scale (0.001 g, Model GF 400, A&D Weighing Co. Ltd., CA, USA), the egg components, including yolk and albumen, were separated with a commercially hand‐held egg separator. A moist cloth napkin was used to get rid of the adhering albumen residues from the yolk, and then it absolutely was weighed. The eggshells were washed with water, dried for 48 h, and weighed (Hossaninejad et al., 2021). Eggshell thickness was measured using a micrometre apparatus (0.001 mm, Model 293‐240; Mitutoyo Co., Ltd., Kanagawa, Japan) at three disparate sites (top, middle, and bottom) were averaged to calculate the overall eggshell thickness. The albumen weight was calculated by subtracting yolk + shell weights from the whole egg weight. Haugh unit, within only 6 h following the egg collection, was calculated based on the following formula (Khatibi et al., 2021):

$$\begin{array}{ccc} \mathrm{Haugh}\;\mathrm{unit}\; & = & \;100\;\times \log\;[\mathrm{albumen}\;\mathrm{height}\;\left(\mathrm{mm}\right)+7.57\\ & & -{\left(1.7\;\times \mathrm{egg}\;\mathrm{weight}\;\left(g\right)\right)}^{0.037}]. \end{array}$$

### 2.5 Bone sampling and analytical methods

At the end of 98 weeks of age, after random selection of one bird from each replicate, the birds were first weighed and then killed by cervical dislocation to measure tibia characteristics. Each bird's left tibia was removed, transferred to sealed plastic bags, and maintained at −20°C for further analysis. Based on the method proposed by the Association of Official Analytical Chemists (Latimer, 2016), the left tibias were first subjected to 5 min boiling to loosen the muscle tissue; then, the meat, connective tissue, and fibula bone were completely removed using scissors and forceps. After cleaning the tibiae, they were placed in an ethanol container (to remove water and polar lipids) for 48 h. The bones were then extracted in anhydrous ether for 24 h (to remove nonpolar lipids). The tibias were dried at 105°C for 24 h and then weighed. The weight of the tibia was recorded. To determine tibia ash content, the ash of the bone was used in a muffle furnace for 18 h at 600°C.

### 2.6 Chemical analysis

The oven drying and Kjeldahl methods were performed according to methods 926.12 and 981.10 of the AOAC International, respectively, for determining DM and CP contents in feed samples (Latimer, 2016). To analyze the dietary Mn content, a sample from each treatment (three replicates/treatment) was ashed at 550°C for 14 h; it was then dissolved in 10 mL of 6 *N* HCl and 30 mL demineralized water at high temperature using a sand heater (300°C for 15 min). After filtration, the volume was increased to 100 mL using demineralized water (Mabe et al., 2003). The Mn content in ash sample was measured using flame atomic absorption spectrophotometer (Perkin Elmer A Analyst 100, Wellesley, MA, USA).

### 2.7 Statistical analysis

All data were analyzed for normality using SAS 9.1 software through the Univariate plot normal procedure; then, the data were analyzed by using the General Linear Model procedure of SAS Institute (SAS, 2003). According to analysis of variance (ANOVA), differences among treatments mean were compared using Tukey test, and the values obtained were considered different at a statistical level of 5%. Orthogonal polynomials for linear and quadratic responses to diet Mn concentration were calculated to explore the relationships between dietary Mn level as independent variables and the respective traits as dependent variables. The dietary Mn concentration for maximum response in performance variables, that is *R* ^2^ was significant, was predicted using the broken‐line regression models, using the nonlinear modelling option in SAS, with the dietary nutrient's density as the independent variable (Robbins et al., 2006). The iterative procedure makes repeated estimates for coefficients and minimizes residual error before the best‐fit line is achieved. To assist in choosing a suitable model, coefficient of determination (*R* ^2^), adjusted *R* ^2^ (adj. *R* ^2^), root means square error (RMSE), and Akaike's information criterion (AIC) values were calculated using the following formulas (Kazemi et al., 2022):

$$Y=L+U\times \left(R-X\right)\times I,$$

$$Y=L+U\times {\left(R-X\right)}^{2}\times I,$$

$$\;R^{2}=\frac{\left(\mathrm{corrected}\;\mathrm{total}\;\mathrm{sum}\;\mathrm{of}\;\mathrm{squares}-\mathrm{sums}\;\mathrm{of}\;\mathrm{squares}\;\mathrm{for}\;\mathrm{error}\right)}{\mathrm{corrected}\;\mathrm{total}\;\mathrm{sum}\;\mathrm{of}\;\mathrm{squares}}\;,$$

$$\begin{array}{ccc} \mathrm{Adjusted}\;R^{2} & = & 1-\left[\frac{\mathrm{sums}\;\mathrm{of}\;\mathrm{squares}\;\mathrm{for}\;\mathrm{error}}{\left(N-1\right)}\right]\\ & & ÷\left[\frac{\mathrm{corrected}\;\mathrm{total}\;\mathrm{sum}\;\mathrm{of}\;\mathrm{squares}}{\left(N-1\right)}\right], \end{array}$$

$$\mathrm{RMSE}=\sqrt{\frac{\sum_{t=1}^{n}{\left(yt-\hat{y}t\right)}^{2}}{N}}\;,$$

$$\mathrm{AIC}=N\times \ln\left(\frac{\mathrm{sums}\;\mathrm{of}\;\mathrm{squares}\;\mathrm{for}\;\mathrm{error}}{N}\right)+2P,$$

where *Y* is the dependent variable, *L* is the theoretical maximum, *R* is the requirement, *X* is the independent variable, *I* = 1 (if *X* < *R*) or *I* = 0 (if *X* > *R*), *U* is the rate constant, *yt* is the observed values, *ŷt* is the predicted values, *N* is the number of observations, *P* = *k* + 1, and *k* is the number of parameters.

A multi‐linear regression model was applied to estimate the relative bio‐efficacy (RBE) value of inorganic Mn (Mn‐oxide and Mn‐sulphate) to Mn‐organic. The nonlinear models procedure (PROC NLIN) of the SAS system was applied, and the models were fit using the following equation:

$$Y=a+b_{1}X_{1}+B_{2}X_{2}+B_{3}X_{3},$$

where *Y* is the dependent variable, *a* is the intercept (parameters with the basal diet), *b* is the asymptote response, *b*_1_ is the slope ratio for Mn‐oxide, *b*_2_ is the slope ratio for inorganic Mn‐sulphate, *b*_3_ is the slope ratio for organic Mn, and *X*_1_, *X*_2,_ and *X*_3_ are dietary supplemented levels of Mn‐oxide, Mn‐sulphate, and organic Mn, respectively.

The RBE value is the ratio between the standard amount and testing source that is required to generate equivalent responses in which a nutrient source at different levels is used and then it is compared to a reference standard based on a biological response such as growth and bone mineralization (Littell et al., 1997). The RBE values for inorganic Mn sources relative to organic Mn were provided by comparing the slope ratios:

$$\mathrm{RBE}=\frac{b_{1}\;\mathrm{and}\;b_{2}}{b_{3}}\;.$$

## 3 RESULT

### 3.1 Egg production performance

The effect of different treatments on egg production performance indices is shown in Table 3. Dietary supplementation with either organic or inorganic Mn significantly enhanced the egg production (EP), egg mass (EM), and feed conversion ratio (FCR) compared with the non‐Mn supplemented diet. Daily feed intake (FI) and egg weight were not influenced by dietary supplementation with organic and inorganic Mn. Productive performance was improved in a linear manner (*p* < 0.05) as an effect of increased dietary Mn concentration with Mn‐oxide. The birds fed supplemented diet at 90 mg/kg Mn‐oxide reached the highest EP, EM, and lowest FCR. However, differences were not significant. An improvement was noticed in egg production performance traits (EP, EM, and FCR) by using diet supplementation with Mn‐organic and Mn‐sulphate up to 60 mg/kg Mn, but by increasing dietary supplementation to higher level (90 mg/kg Mn), birds showed undesirable performance (quadratic effect, *p* < 0.05).

**TABLE 3:** Effect of dietary supplementation with different sources and levels of manganese (Mn) on egg production performance of aged laying hens (87–98 weeks of age)^a^.

	Dietary supplementation Mn source and level, mg/kg											Mn dose response, *p*‐Value					
	Non‐Sup.	Mn‐oxide			Mn‐sulphate			Mn‐organic				Mn‐oxide		Mn‐sulphate		Mn‐organic	
Age, week		30	60	90	30	60	90	30	60	90	SEM	L	Q	L	Q	L	Q
	Egg production, %																
87–90	73.87^b^	78.33^ab^	80.28^a^	83.61^a^	82.96^a^	84.44^a^	78.12^ab^	81.92^a^	81.30^a^	80.65^a^	2.70	0.01	0.83	0.28	0.02	0.02	0.05
91–94	74.81^b^	77.51^ab^	78.85^a^	78.98^a^	79.15^a^	79.39^a^	79.63^a^	81.19^a^	80.65^a^	80.42^a^	2.07	0.17	0.56	0.20	0.42	0.04	0.10
95–98	62.57^b^	64.41^ab^	64.67^ab^	64.72^ab^	70.11^a^	69.79^a^	70.93^a^	72.13^a^	73.61^a^	71.62^a^	1.65	0.37	0.59	0.01	0.08	0.01	0.01
Overall	70.42^b^	73.42^ab^	74.60^ab^	75.77^ab^	77.41^a^	77.87^a^	76.23^a^	78.41^a^	78.52^a^	77.56^a^	1.67	0.03	0.61	0.01	0.03	0.01	0.01
	Egg weight, g/egg																
87–90	66.42	66.23	67.64	65.63	65.60	66.36	66.67	66.33	66.17	66.37	0.78	0.81	0.32	0.63	0.42	0.82	0.83
91–94	65.11	65.07	64.95	65.60	65.40	64.46	64.90	64.61	63.54	64.73	0.53	0.62	0.57	0.58	0.88	0.18	0.23
95–98	60.05	55.52	54.23	55.42	58.11	57.23	58.43	58.88	57.83	56.25	1.55	0.03	0.08	0.40	0.31	0.60	0.85
Overall	63.86	62.27	62.27	62.22	63.04	62.67	63.33	63.27	62.51	62.45	0.57	0.04	0.14	0.48	0.23	0.16	0.51
	Egg mass, g/b/d																
87–90	49.11^b^	51.86^ab^	54.27^a^	54.89^a^	54.43^a^	56.03^a^	52.03^ab^	54.33^a^	53.77^a^	53.56^a^	1.84	0.03	0.57	0.01	0.03	0.04	0.10
91–94	48.66	50.44	51.21	51.83	51.78	51.16	51.66	52.45	51.25	52.04	1.38	0.12	0.69	0.29	0.44	0.12	0.24
95–98	37.58^b^	35.68^b^	35.11^b^	35.81^b^	40.71^a^	39.93^a^	41.49^a^	42.49^a^	42.66^a^	40.34^a^	1.48	0.28	0.29	0.11	0.60	0.02	0.03
Overall	45.11^b^	45.99^ab^	46.87^ab^	47.51^ab^	48.97^a^	49.04^a^	48.40^a^	49.76^a^	49.23^a^	48.65^a^	1.06	0.09	0.90	0.02	0.06	0.01	0.02
	Feed intake, g/b																
87–90	116.33	115.50	116.58	115.38	116.11	115.34	114.74	116.60	115.93	116.20	1.50	0.54	0.77	0.65	0.62	0.66	0.95
91–94	116.45	114.54	116.27	114.07	115.65	114.14	113.03	115.69	115.27	115.56	1.65	0.25	0.88	0.42	0.83	0.42	0.54
95–98	115.27	114.17	115.41	114.03	114.95	113.84	113.15	116.05	114.88	115.19	1.61	0.56	0.87	0.51	0.68	0.68	0.75
Overall	116.01	114.74	116.09	114.49	115.57	114.44	113.64	116.11	115.36	115.65	1.57	0.39	0.85	0.45	0.71	0.54	0.88
	Feed conversion ratio, g FI/g EM																
87–90	2.385^a^	2.250^ab^	2.155^ab^	2.111^ab^	2.154^ab^	2.064^b^	2.220^ab^	2.147^ab^	2.162^ab^	2.183^ab^	0.08	0.03	0.62	0.02	0.05	0.04	0.09
91–94	2.407	2.278	2.271	2.211	2.253	2.237	2.192	2.206	2.255	2.223	0.07	0.06	0.61	0.07	0.50	0.08	0.18
95–98	3.079	3.209	3.305	3.199	2.842	2.856	2.753	2.744	2.717	2.873	0.11	0.16	0.25	0.05	0.52	0.02	0.04
Overall	2.579	2.499	2.480	2.417	2.370	2.336	2.353	2.334	2.353	2.378	0.06	0.07	0.88	0.01	0.07	0.01	0.02

*Note*: Values with different superscripts within a row are significantly different (*p* < 0.05).

Abbreviation: Non‐Sup., non‐supplemented.

^a^ Every mean is the average of six replicates with 12 birds each (72 birds per treatment).

### 3.2 Eggshell quality

The effects of dietary supplementation with Mn on egg quality characteristics are presented in Table 4. Relative eggshell weight was affected as dietary Mn supplementation levels increased by Mn‐organic and Mn‐sulphate (quadratic effect, *p* < 0.05) and Mn‐oxide (liner effect, *p* < 0.05), while egg shape index, the other eggshell quality traits, such as egg special gravity and shell thickness showed non‐significant response (*p* > 0.05) to dietary Mn supplementation level. Similarly, the internal and external egg quality traits such as shape index, Hugh unit, and egg composition showed a non‐significant response (*p* > 0.05) to dietary Mn supplementation (data not shown).

**TABLE 4:** Effect of dietary supplementation with different source and level of manganese (Mn) on egg and bone quality characteristic of aged laying hens (87−98 weeks of age)^a^.

	Dietary supplementation Mn source and level, mg/kg											Mn dose response, *p*‐value					
	Non‐Sup.	Mn‐oxide			Mn‐sulphate			Mn‐organic				Mn‐oxide		Mn‐sulphate		Mn‐organic	
Age, week		30	60	90	30	60	90	30	60	90	SEM	L	Q	L	Q	L	Q
	Eggshell weight, g/100 g egg weight																
87–90	8.92^b^	8.94^b^	9.23^ab^	9.20^ab^	9.48^ab^	9.45^ab^	9.27^ab^	9.53^a^	9.75^a^	9.52^a^	0.15	0.01	0.80	0.01	0.01	0.01	0.01
91–94	8.72^b^	8.89^b^	9.32^ab^	9.24^ab^	8.88^b^	9.74^ab^	8.96^ab^	9.49^ab^	9.91^a^	9.20^ab^	0.32	0.01	0.44	0.01	0.03	0.04	0.01
95–98	8.70^b^	9.32^ab^	9.43^ab^	9.36^ab^	8.85^b^	9.78^a^	9.35^ab^	9.66^a^	9.69^a^	9.26^ab^	0.40	0.04	0.13	0.04	0.19	0.12	0.01
Overall	8.78^b^	9.05^ab^	9.33^ab^	9.26^ab^	9.07^ab^	9.65^ab^	9.19^ab^	9.56^a^	9.78^a^	9.33^ab^	0.17	0.01	0.10	0.01	0.01	0.01	0.01
	Eggshell thickness, μm																
87–90	394	392	392	409	403	396	405	396	413	402	11.10	0.09	0.14	0.43	0.99	0.12	0.97
91–94	386	375	378	389	375	389	383	384	392	381	9.22	0.67	0.10	0.27	0.68	0.28	0.77
95–98	361	386	374	355	372	372	366	377	373	367	10.05	0.53	0.37	0.08	0.10	0.80	0.55
Overall	380	384	383	384	383	385	384	385	388	383	6.66	0.38	0.12	0.38	0.53	0.16	0.40
	Egg special gravity, g/cm^3^																
87–90	1.082	1.083	1.084	1.082	1.081	1.082	1.082	1.082	1.085	1.083	0.003	0.88	0.53	0.72	0.79	0.15	0.82
91–94	1.080	1.080	1.080	1.080	1.077	1.081	1.080	1.080	1.085	1.082	0.003	0.47	0.69	0.39	0.83	0.13	0.21
95–98	1.085	1.087	1.080	1.085	1.085	1.083	1.084	1.083	1.086	1.085	0.005	0.92	0.54	0.28	0.95	0.82	0.77
Overall	1.082	1.083	1.084	1.082	1.081	1.082	1.082	1.082	1.085	1.083	0.003	0.88	0.53	0.72	0.79	0.15	0.82
	Haugh unit																
87–90	94.32	88.83	90.43	91.10	91.20	92.94	90.42	85.43	90.12	90.07	3.01	0.29	0.17	0.16	0.16	0.11	0.11
91–94	94.82	90.19	90.16	92.57	91.92	94.70	90.30	90.16	90.51	92.74	2.53	0.22	0.11	0.18	0.18	0.13	0.14
95–98	93.11	89.31	89.35	93.23	92.77	93.45	90.15	91.09	89.99	91.79	3.99	0.96	0.09	0.40	0.49	0.36	0.42
Overall	94.09	89.44	89.98	92.30	91.96	93.70	90.29	88.89	90.21	91.53	2.33	0.31	0.11	0.19	0.60	0.13	0.14

*Note*: Values with different superscripts within a row are significantly different (*p* < 0.05).

Abbreviation: Non‐Sup., non‐supplemented.

^a^ Every mean is the average of six replicates and six eggs for each replicate (36 eggs for each treatment were measured).

### 3.3 Tibia bone characteristic

The influence of dietary sources and levels of Mn on the tibia bone characteristic of laying hens at 98 weeks of age is shown in Table 5. The ANOVA analysis indicates that all tibia bone characteristic parameters did not exhibit a significant response to the dietary treatments (*p* > 0.05). We did not observe differences in tibia Mn, calcium, and phosphorus content (data not shown) between the dietary treatments (*p* > 0.05).

**TABLE 5:** Effect of dietary supplementation with different source and level of manganese on bone quality of aged laying hens (87−98 weeks of age) measured at the end of experiment^a^.

	Dietary supplementation Mn source and level, mg/kg											Mn dose response, *p*‐value					
	Non‐Sup.	Mn‐oxide			Mn‐sulphate			Mn‐organic				Mn‐oxide		Mn‐sulphate		Mn‐organic	
Items		30	60	90	30	60	90	30	60	90	SEM	L	Q	L	Q	L	Q
Dry mater	5.24	5.28	5.19	5.22	5.45	5.51	5.29	5.39	4.98	4.82	0.21	0.87	0.96	0.84	0.36	0.09	0.48
Ash	41.89	42.21	43.39	42.43	42.32	42.15	42.33	42.07	45.91	43.76	0.96	0.66	0.74	0.88	0.98	0.10	0.35
Organic matter	58.02	57.79	56.61	57.57	57.82	57.85	57.67	57.93	54.09	56.25	0.97	0.48	0.56	0.64	0.98	0.17	0.35
Ash/organic matter	0.72	0.73	0.77	0.74	0.73	0.72	0.73	0.73	0.87	0.78	0.03	0.42	0.49	0.73	0.89	0.12	0.34

Abbreviation: Non‐Sup., non‐supplemented.

### 3.4 Estimated Mn requirement

**FIGURE 1:**
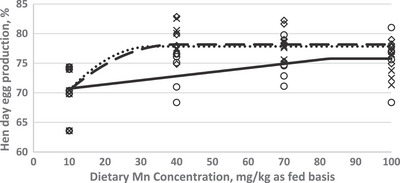
Fitted broken‐line plot of hen‐day egg production (%) of Hy‐Line‐W36 aged laying hens (87–98 weeks of age) as a function of diet manganese (Mn) concentration (mg/kg as fed basis). ○: Liner broken‐line fitted model for Mn‐oxide supplementation, *Y* = 75.77 − 0.07 (82 − *X*) × *I*, *I* = 1 (if *X* < 82 or *I* = 0 (if *X* > 82), *p* < 0.015, *R* ^2^ = 0.23, the break point occurred at 82 ± 18.5. ◊: Quadratic broken‐line fitted model for Mn‐sulphate supplementation, *Y* = 78.17 − 0.009 (40 − *X*)^2^ × *I*, *I* = 1 (if *X* < 40 or *I* = 0 (if *X* > 40), *p* < 0.001, *R* ^2^ = 0.57, the break point occurred at 40 ± 7.5. ×: Quadratic broken‐line fitted model for Mn‐organic supplementation, *Y* = 77.84 – 0.014 (33 − *X*)^2^ × *I*, *I* = 1 (if *X* < 33 or *I* = 0 (if *X* > 33), *p* < 0.001, *R* ^2^ = 0.54, the break point occurred at 33 ± 9.50.

**FIGURE 2:**
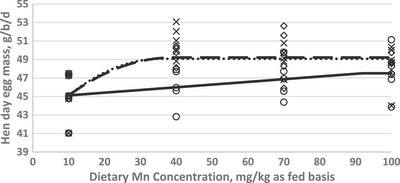
Fitted broken‐line plot of hen‐day egg mass (g/b/d) of Hy‐Line‐W36 aged laying hens (87–98 weeks of age) as a function of diet manganese (Mn) concentration (mg/kg as fed basis). ○: Liner broken‐line fitted model for Mn‐oxide supplementation, *Y* = 47.51 − 0.029 (92 − *X*) × *I*, *I* = 1 (if *X* < 92 or *I* = 0 (if *X* > 92), *p* < 0.023, *R* ^2^ = 0.23, the break point occurred at 92 ± 15.3. ◊: Quadratic broken‐line fitted model for Mn‐sulphate supplementation, *Y* = 49.21 − 0.005 (39 − *X*)^2^ × *I*, *I* = 1 (if *X* < 39 or *I* = 0 (if *X* > 39), *p* < 0.002, *R* ^2^ = 0.43, the break point occurred at 39 ± 8.5. ×: Quadratic broken‐line fitted model for Mn‐organic supplementation, Y = 90.13 − 0.006 (36 − *X*)^2^ × *I*, *I* = 1 (if *X* < 36 or *I* = 0 (if *X* > 36), *p* < 0.002, *R* ^2^ = 0.28, the break point occurred at 36 ± 9.5.

**FIGURE 3:**
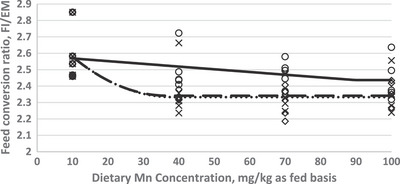
Fitted broken‐line plot of feed conversion ratio (g FI/ g EM) of Hy‐Line‐W36 aged laying hens (87–98 weeks of age) as a function of diet manganese (Mn) concentration (mg/kg as fed basis). ○: Liner broken‐line fitted model for Mn‐oxide supplementation, *Y* = 2.437 + 0.002 (89 − *X*) × *I*, *I* = 1 (if *X* < 89 or *I* = 0 (if *X* > 89), *p* < 0.035, *R* ^2^ = 0.33, the break point occurred at 89 ± 15.5. ◊: Quadratic broken‐line fitted model for Mn‐sulphate supplementation, *Y* = 2.36 − 0.0003 (38 − *X*)^2^ × *I*, *I* = 1 (if *X* < 38 or *I* = 0 (if *X* > 38), *p* < 0.001, *R* ^2^ = 0.45, the break point occurred at 38 ± 11.6. ×: Quadratic broken‐line fitted model for Mn‐organic supplementation, *Y* = 2.33 − 0.0003 (39 − *X*)^2^ × *I*, *I* = 1 (if *X* < 39 or *I*=0 (if *X* > 39), *p* < 0.001, *R* ^2^ = 0.54, the break point occurred at 40.0 ± 10.5.

In order to investigate the effect of dietary Mn supplementation on egg performance traits, a crucial goal of the current study was to estimate Mn requirement of Hy‐Line W‐36 aged laying hens. The optimization model was solved by using the NLIN program SAS 9.1 procedure. Fitted broken‐line models for the egg performance traits as a function of dietary Mn concentration are shown in Figures 1, 2, 3. Based on the highest adj. *R* ^2^ and the lowest RMSE and AIC, the linear broken‐line (LBL) fit model was selected to estimate Mn requirements for the diets supplemented with Mn‐oxide and the quadratic broken‐line fit model was achieved to estimate the Mn requirement for the diet supplemented with Mn‐sulphate and or Mn‐organic. The best balance of performance traits and dietary Mn concentration was found at 82–92 mg/kg of diet as supplemented by Mn‐oxide, 38–40 mg/kg of diet as supplemented by Mn‐sulphate, and 33–40 mg/kg of diet as supplemented by Mn‐organic for egg production performance traits (EP, EM, and FCR).

### 3.5 Relative bio‐efficacy of Mn sources

The slope ratio method for the variables of performance and eggshell quality response was employed to measure the RBE of inorganic Mn (Mn‐oxide and Mn‐sulphate) compared with Mn‐organic (Table 6). In the current study, we observed a few eggshell quality parameters, such as eggshell relative weight, that exhibited a significant response to the Mn dietary supplement levels and sources (*p* < 0.05), and it is reasonable to apply these sensitive parameters to evaluate the RBE of organic Mn compared with inorganic Mn in aged layer eggshell quality. The RBE of inorganic Mn‐oxide was estimated 45%, 30%, 36%, and 37% as efficacious as Mn‐organic at equimolar Mn levels for EP, EM, FCR, and RESW traits, respectively. Similarly, the RBE of Mn‐sulphate was estimated 87%, 94%, 99%, and 78% as efficacious as Mn‐organic at equimolar Mn levels for EP, EM, FCR, and RESW traits, respectively.

**TABLE 6:** Parameters of linear model describe the relationship between response criteria and supplementary levels and relative bio‐efficacy values of manganese (Mn) source.

Criterion response	Y‐intercept	Estimated *b* coefficient for Mn source			*p*‐Value	*R* ^2^	Relative bio‐efficacy, %		
		Mn‐oxide	Mn‐sulphate	Mn‐organic			Mn‐oxide	Mn‐sulphate	Mn‐organic
Egg production, %	72.52	0.0353	0.0684	0.0786	0.001	0.26	45	87	100
Egg mass, g/b/d	46.18	0.0122	0.0380	0.0406	0.003	0.21	30	94	100
Feed conversion ratio	2.520	−0.0009	−0.0025	−0.0025	0.001	0.26	36	99	100
Relative eggshell weight, %	9.09	0.0023	0.0047	0.0062	0.010	0.20	37	76	100

*Note*: The egg productive performance criteria for all date were analyzed by multi‐linear regression using; *Y* = *a* + *b*1*X*1 + *b*2*X*2 + *b*3*X*3.

## 4 DISCUSSION

### 4.1 Egg production performance

This study indicates that dietary Mn supplementation levels and sources affect egg production and feed efficiency, which is consistent with previous studies. It has been shown that fed diet supplemented with Mn can improve egg production performance in laying hens (Fassani et al., 2000; Hossain & Bertechini, 1998; Mabe et al., 2003; Xiao et al., 2015, 2014). The effects of graded levels of Mn (25, 50, and 75 mg/kg) on performance of commercial layers were evaluated during the period from 42 to 52 weeks of age (Hossain & Bertechini, 1998). When egg production and egg weight data were regressed against the level of Mn in the diet, a quadratic response for egg production and a linear response for egg weight were obtained. The highest egg production was observed with supplementation of 50 mg/kg Mn, whereas the highest egg weight was observed with 75 mg/kg of Mn in the diet. Xiao et al. (2015) in an experiment that was conducted for bio‐efficacy comparison of organic Mn with inorganic Mn (0, 25, 50, 100, and 200 mg/kg) in Hy‐Line Brown laying hens reported that dietary Mn supplementation with organic Mn at 50 mg/kg level had the highest feed efficiency and the lowest feed intake, when the data were extrapolated from the 12‐week feeding trial (55–62 weeks of age). In contrast with the current study and above reports, many studies (Faria et al., 1999; Mabe et al., 2003; Sazzad et al., 1994; Venglovska et al., 2014; Xiao et al., 2015, 2014; Yıldız et al., 2011) reported that egg performance parameters did not exhibit a significant response to Mn dietary supplementation levels and sources (*p* > 0.05). Sazzad et al. (1994) observed that the performance parameters of 23‐ to 35‐week‐old laying hens were not affected by dietary supplementation of Mn at 20, 40, or 80 mg/kg. Similarly, Yildiz et al. (2011) reported that the dietary supplementation of Mn (35 or 70 mg/kg) did not affect the performance of laying hens between 22 and 42 weeks of age. Faria et al. (1999) showed that dietary Mn levels (70, 140, or 210 mg/kg) did not affect the performance of laying hens as did Venglovska et al. (2014), who stated that feeding 120 mg/kg did not affect the performance of 20‐week‐old hen layers. In an experiment conducted by Yildiz et al. (2011), dietary addition of Mn (15‐70 mg/kg), regardless of its form, did not affect performance, but egg weight was increased by supplementation with organic Mn (Mn‐Bioplex) in laying hens at 49 weeks of age. In the current study, the basal diet Mn content was 10 mg/kg, and the age of the birds was 87–98 weeks; it is reasonable that the effect of adding Mn to aged layers was more apparent than young layers. On the other hand, lowering the Mn content in the basal diet should reinforce the dose‐response to Mn dietary supplementation. Mn is one of the enzyme cofactors involved in the synthesis of cholesterol, which is the main structure of ovarian steroids (Olgun, 2017). Dietary deficiency of Mn caused changes in circulating ovarian steroids in laying hens, and consequently reduced reproduction in hens (Feng & Feng, 1998).

### 4.2 Eggshell quality

As shown in Table 4, eggshell relative weight was significantly improved by the dietary supplementation of Mn in aged laying hens. These results are in correspondence with other reports that have shown that fed diet supplemented with Mn can improve eggshell strength in laying hens (Fassani et al., 2000; Mabe et al., 2003; Xiao et al., 2014). Xiao et al. (2015) showed that as an organic source, Mn‐amino acid complexes improved eggshell quality parameters in aged laying hens compared to inorganic Mn‐sulphate (Xiao et al., 2015). Conversely, other studies comparing the availability of inorganic with organic sources of Mn have reported no significant difference in either performance or eggshell quality in laying hens (Swiatkiewicz & Koreleski, 2008; Yildiz et al., 2011). The positive impacts of organic microelements in laying hens’ nutrition and egg quality characteristics were documented by Gheisari et al. (2011) who demonstrated that inclusion of organic Zn, Mn, and Cu in corn‐soybean diets was able to improve eggshell and egg albumen qualities (Gheisari et al., 2011). In addition, Favero et al. (2013) concluded that the replacement of inorganic sulphates of Zn, Mn, and Cu with organic amino acid complexes of these microelements in broiler breeders enhanced eggshell quality (i.e., eggshell weight, and thickness) and reduced early embryo mortality (Favero et al., 2013). It is well known that Mn from organic or inorganic sources could affect the mechanical properties of eggshell by affecting the calcite crystal formation and adjusting crystal holographic structure of the eggshell (Mabe et al., 2003; Swiatkiewicz & Koreleski, 2008). Mn is an activator of enzymes that participated in the synthesis of mucopolysaccharides and glycoproteins, which participate in shell organic matrix formation (Saleh et al., 2020).

### 4.3 Tibia bone characteristic

The Mn supplementation levels and sources in the present study did not affect the tibia bone quality traits (Table 5) and composition such as phosphorus, iron, zinc, Mn, and magnesium contents of the tibia bone (data not shown). However, it has been proved that Mn plays an important role in bone formation (Olgun, 2017) and in many biochemical processes by activating enzymes, such as pyruvate carboxylase, superoxide dismutase, and glycosyltransferase (Suttle, 2010). The current experiment's results indicate that bone properties in older hens are not sensitive to the dietary Mn source and level. There is a lack of literature data on the effect of dietary Mn on the bone quality in laying hens (Wang et al., 2002).

### 4.4 Estimated Mn requirement

The current experiment's data indicated that a certain level of dietary Mn (30–40 mg/kg when supplemented by Mn‐sulphate or Mn‐organic and 80–90 mg/kg when supplemented by Mn‐oxide) concentration results in an optimum egg productive performance. Various diet Mn levels for laying hens have been reported by researchers. The NRC (1994) proposed 20 mg/kg Mn concentration in laying hens’ diet. The Mn requirement for maximal egg production performance traits in laying hens, as a result of improvement in genetics, management, and nutrition over the past 50–60 years, is estimated to be higher than NRC levels. For instance, the Mn requirement of laying hens was recommended as 50 mg/kg (Ochrimenko et al., 1992), 60 mg/kg (Leeson & Summers, 2009), 105 mg/kg (Sazzad et al., 1994), and 120 mg/kg for laying hens (Fassani et al., 2000). In contrast to the results of the current experiment, dietary Mn addition was not necessary for the basal diet (14 mg/kg Mn) of 22‐ to 42‐week‐old laying hens (Yildiz et al., 2011). The dietary requirements for Mn depend on the criteria used in studies. For example, a total of 25 mg/kg Mn is considered sufficient to support maximum egg production, egg weight, and FCR, but for maximal eggshell quality, the minimal requirement for laying hens is between 50 and 100 mg/kg (Yang et al., 2012). Additionally, reported the Mn requirement may vary with the type of diet and supplement used (NRC, 1994). The bioavailability of Mn is very low in most practical feedstuffs, and there is evidence that practical ingredients reduce the bioavailability of inorganic dietary Mn (Halpin & Baker, 1986). For example, the Mn requirement 14 mg/kg reported (Southern & Baker, 1983) for chicks fed a semi‐purified dextrose‐casein diet is much lower than 50–70 mg/kg for growth and bone development in chicks (Caskey et al., 1939), pekin ducks (Wu & Shen, 1978), turkeys (Kealy & Sullivan, 1966), and pheasants (Scott et al., 1959) fed with diet containing practical ingredients. The same levels (60 mg/kg) were reported as the requirement for adult turkeys (Atkinson et al., 1976), pheasants, and quails (NRC, 1994). Overall, the current study's result confirms that the Mn requirement (25 mg/kg) suggested by NRC (1994) is inadequate for aged layers.

### 4.5 Relative bio‐efficacy of Mn sources

In the current experiment, the RBE of Mn‐oxide and Mn‐sulphate was estimated to be 30%–45% and 76%–99%, respectively, as efficacious as Mn‐organic at equimolar Mn levels. However, limited reports are available on the RBE of different Mn sources for laying hens, and certain published results are inconsistent (Attia et al., 2010; Fernandes et al., 2008; Xiao et al., 2015). The RBE of organic Mn source depends on its absorption, and inorganic Mn source depends on its solubility in the intestine (Olgun, 2017). It is generally accepted that the organic trace element is advantageous for absorption and, thus, causes less environmental contamination (Ao et al., 2006; Li et al., 2005; mHy‐Line, 2020; Xiao et al., 2015; Yan & Waldroup, 2006). As an Mn source, Mn‐sulphate is reported to be more available than Mn‐oxide and Mn‐carbonate (Black et al., 1984; Korol et al., 1996). Similarly, it was noted that the dietary addition of Mn‐sulphate instead of Mn‐oxide improved performance parameters such as feed intake and feed conversion ratio of laying hens and decreased the percentage of broken eggs (Gheisari et al., 2011).

## 5 CONCLUSION

The findings of the current study demonstrate that dietary Mn supplementation with either organic or inorganic Mn can improve the performance and eggshell quality in aged laying hens. Based on the broken line regression models, the performance trait was optimized at 30–40 mg/kg dietary Mn concentration when supplemented by Mn‐sulphate or Mn‐organic. However, it was 80–90 mg/kg when supplemented by Mn‐oxide. The RBE of inorganic Mn sources include Mn‐oxide and Mn‐sulphate in comparison with organic source (Mn‐organic) were estimated 45% and 87% (for EP trait), 30% and 94% (for EM trait), 36% and 99% (for FCR trait), and 37% and 78% (for RESW trait). Overall, in the aged laying hens, Mn requirement is higher than the NRC's (1994) recommendation, and feed supplementation with organic source is more effective than Mn‐oxide.

## AUTHOR CONTRIBUTIONS

Heydar Zarghi and Nafise Barzegar designed and carried out the experimental trail. Nafise Barzegar performed lab analysis. Heydar Zarghi performed the statistics, tabulated the data, and wrote the draft paper. Ahmad Hassanabadi revised and reviewed the manuscript.

## CONFLICT OF INTEREST STATEMENT

The authors declare no conflict of interest. The authors are faculty member at Ferdowsi University of Mashhad, Mashhad, Iran. They are employed by an institutional that has a primary function research and/or education. The authors did not submit this manuscript as an official representative or on behalf of the government.

### PEER REVIEW

The peer review history for this article is available at https://publons.com/publon/10.1002/vms3.1116.

## ETHICS STATEMENT

The authors confirm that the ethical policies of the journal, as noted in the journal's author guidelines page, have been adhered to and the appropriate ethical review committee approval has been received. The authors also confirm that they have followed EU standards for the protection of animals used for scientific purposes and feed legislation.

## Data Availability

The data that support the findings of this study are included in this published article.
